# From ear to body: the auditory-motor loop in spatial cognition

**DOI:** 10.3389/fnins.2014.00283

**Published:** 2014-09-05

**Authors:** Isabelle Viaud-Delmon, Olivier Warusfel

**Affiliations:** ^1^CNRS, UMR 9912, Sciences et Technologies de la Musique et du SonParis, France; ^2^Institut de Recherche et Coordination Acoustique/Musique, UMR 9912, Sciences et Technologies de la Musique et du SonParis, France; ^3^Sorbonne Universités, Université Pierre et Marie Curie, UMR 9912, Sciences et Technologies de la Musique et du SonParis, France

**Keywords:** spatial audition, Morris water maze, auditory landmarks, virtual reality, navigation, spatial memory, allocentric representation, auditory scene

## Abstract

Spatial memory is mainly studied through the visual sensory modality: navigation tasks in humans rarely integrate dynamic and spatial auditory information. In order to study how a spatial scene can be memorized on the basis of auditory and idiothetic cues only, we constructed an auditory equivalent of the Morris water maze, a task widely used to assess spatial learning and memory in rodents. Participants were equipped with wireless headphones, which delivered a soundscape updated in real time according to their movements in 3D space. A wireless tracking system (video infrared with passive markers) was used to send the coordinates of the subject's head to the sound rendering system. The rendering system used advanced HRTF-based synthesis of directional cues and room acoustic simulation for the auralization of a realistic acoustic environment. Participants were guided blindfolded in an experimental room. Their task was to explore a delimitated area in order to find a hidden auditory target, i.e., a sound that was only triggered when walking on a precise location of the area. The position of this target could be coded in relationship to auditory landmarks constantly rendered during the exploration of the area. The task was composed of a practice trial, 6 acquisition trials during which they had to memorize the localization of the target, and 4 test trials in which some aspects of the auditory scene were modified. The task ended with a probe trial in which the auditory target was removed. The configuration of searching paths allowed observing how auditory information was coded to memorize the position of the target. They suggested that space can be efficiently coded without visual information in normal sighted subjects. In conclusion, space representation can be based on sensorimotor and auditory cues only, providing another argument in favor of the hypothesis that the brain has access to a modality-invariant representation of external space.

## Introduction

We perceive the world around us through multiple senses. When we explore an environment, we produce idiothetic information through vestibular receptors, muscle and joint receptors, and efference copy of commands that generate movement. Visual, auditory, and olfactory stimuli caused by movement can also be used to encode our spatial environment. However, spatial cognition has mainly been studied in experimental situations without auditory information: View-based approaches for spatial memory are the most common. For example, with very few exceptions (e.g., Loomis et al., 1998, 2001; Afonso et al., 2010), landmark based navigation has been studied in vision.

The Morris water maze test is a classical paradigm used to evaluate spatial learning in animal models (Morris, 1981, 1984). It requires the animal to locate a hidden platform, using available room cues, which is submerged below the surface of a large circular arena filled with opaque water. The manipulation of available visual information allows for the determination of the types of cues that are used to solve the task. The Morris water maze has been widely used to investigate which brain structures are involved in spatial memory, and has largely contributed to the discovery of “place cells.” Place cells fire when an animal is at a specific location in an environment, providing a stable representation, independent of orientation, of the animal's location.

The Morris water task has been adapted to humans in real settings (e.g., Bohbot et al., 2002) and in virtual environments. Virtual reality analogues have been developed and tested in humans for more than 15 years (e.g., Jacobs et al., 1998; Moffat et al., 1998; Sandstrom et al., 1998; Astur et al., 1998; Hamilton and Sutherland, 1999; Chamizo et al., 2003), all of which centered on the visual modality. As in the water maze, participants are required to find a platform (hidden target) surrounded by a set of landmarks. In rodents, few studies have integrated the auditory modalities in their Morris water maze tasks (Rossier et al., 2000; Watanabe and Yoshida, 2007). Likely due to the difficulty in mastering the acoustic parameters of an experimental environment not conceived for auditory experiments, the results of these studies are not convincing.

We created a virtual sound scene composed of landmarks surrounding a hidden target. We asked participants to actively explore this scene in order to learn to locate the target on the basis of cues provided by the auditory-motor loop. It is known that to localize sound requires the integration of multisensory information and the processing of self-generated movements, therefore a stable representation of an auditory source has to be based on acoustic inputs and their relation to motor states (Aytekin et al., 2008). Here we hypothesized that auditory and motor cues would constitute relevant enough information to build a spatial representation of the scene in the absence of any visual cue.

We devised tests to ascertain which aspects of the organization of the landmarks were involved in determining the locus of search and to understand whether the principles of spatial cognition that have been largely developed on the basis of vision hold as general principles independent of the sensory modality or, conversely, are completely dependent on the stimulated sensory modality.

After the acquisition phase, we first investigated whether the most proximal auditory landmark was used to find the target. In a second test, we maintained the adjacency relations of the landmarks but modified their distance with the target. In a third test, we altered the adjacency relationship between landmarks from those that were learned, creating a conflict between landmark location (“where”) and landmark type (“what”). Alterations were made such that one landmark location in the testing configuration maintained identical distance relationships as they were in the learning configuration. In a last test, the boundaries of the surface layout were modified.

It has been suggested that geometric cues are processed separately from non-geometric cues (e.g., Wang and Spelke, 2000; Cheng, 2008), and that different brain activations are associated with boundary-related locations and landmark-related locations (Doeller et al., 2008). There is also a segregation between auditory cortical pathways for the identification and localization of objects (e.g., Rauschecker and Tian, 2000; Ahveninen et al., 2013). We thought that altering the identity of a landmark independently of its location might be a way to distinguish object-related patterns (what) from spatial patterns (where). We therefore expected that the difficulties the participants encountered would be different in function of modifications of the geometrical configuration of the landmarks (like in test 1—Removal and test 2—Rotation), the identity of the landmarks (as in test 3—Switch), or the boundary of the surface layout (as in test 4—Perimeter).

## Materials and methods

### Participants

Eleven participants (6 females and 5 males; 26.7 ± 4.1 years old) took part in the experiment. All were healthy and reported normal hearing. The study was carried out in accordance with the Declaration of Helsinki. After an explanation of the procedure, all participants signed informed consent releases.

### Experimental setup

The participants were equipped with wireless headphones, which delivered an auditory virtual environment updated in real time in accordance with their movements in 3D space. A wireless tracking system (video infrared with passive markers) was used to send the coordinates of the subject's head to the sound rendering system with a refreshing rate of 60 Hz. We used the Spat~ sound rendering engine (Jot and Warusfel, 1995; Jot, 1999) and the ListenSpace auditory scene authoring tool (Delerue and Warusfel, 2002), both developed at Ircam. The rendering system used advanced HRTF-based synthesis of directional cues and room acoustic simulation for the auralization of a realistic acoustic environment. Moving in the virtual environment would for instance modify the level and direction of the direct sound and of the first reflections of each source landmark according to its position and orientation relative to the participant's head. Participants were selected among people whose HRTFs had been previously measured to constitute the Listen HRTF database (http://recherche.ircam.fr/equipes/salles/listen/). Hence, the auditory virtual environment could be rendered binaurally using their individual HRTFs.

#### Exploration area and auditory virtual environment

**Figure 1:**
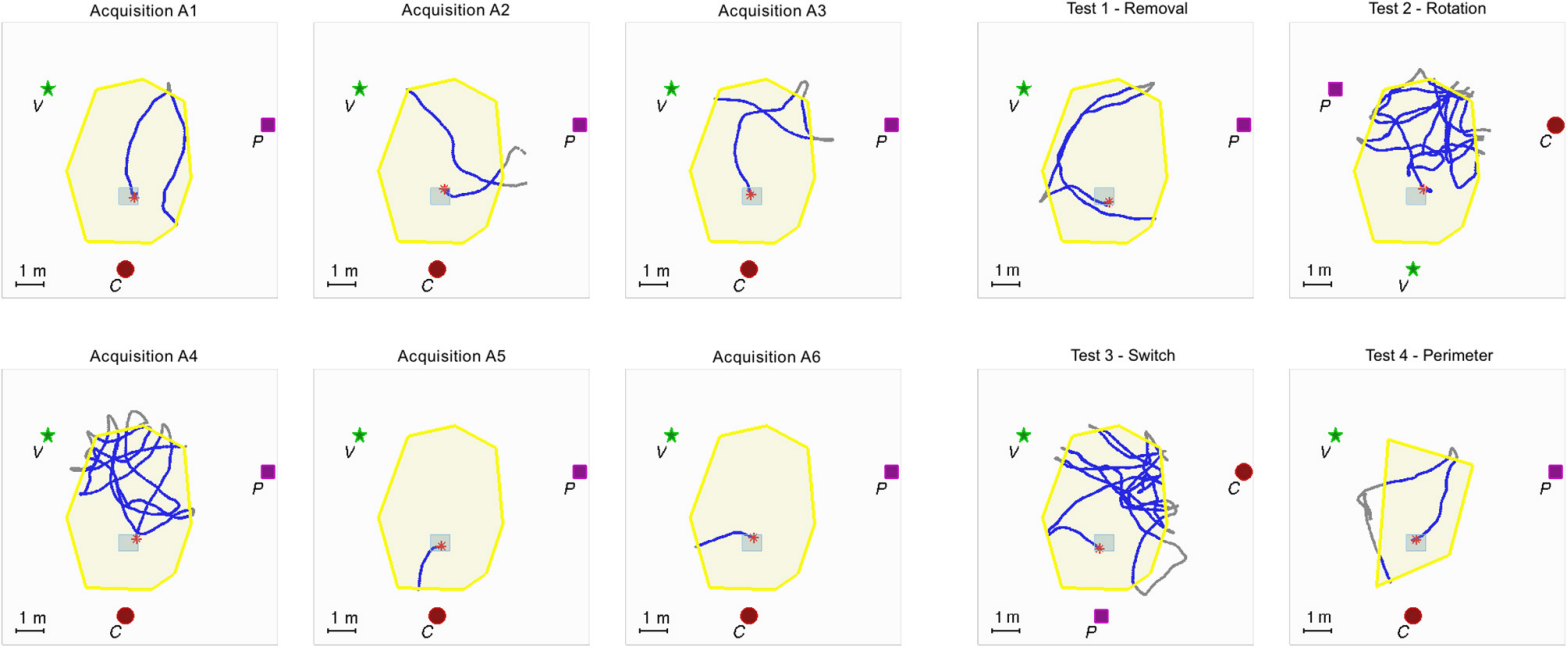
**Example of individual data for one participant**. The experimental setup is composed of 3 auditory landmarks (red spot, green star and purple square, respectively labeled C for cicada, V for voice, and P for piano) surrounding the exploration zone (area = 22 m^2^) delineated by the yellow auditory border (wind heard when outside); “hidden” auditory target (gray squared frame; 40 cm^2^) triggering a whistle tone. The same configuration was maintained for the six acquisition trials, labeled from A1 to A6 (left). During the four test trials (right), the auditory landmarks configuration or the shape of the exploration area were modified.

The experimental room was 25 × 17 m^2^ in size. The auditory virtual environment consisted of 3 distinct landmarks, a target location within the triangle formed by the 3 landmarks, and a delimitated exploration area (see Figure 1). Because the landmarks were distinct, semantic information about landmark identity was available.

The landmarks were located in the periphery of a zone covering a surface of 22 m^2^, the exploration area, centered in the experimental room. The following three familiar and distinct sound samples were used as constantly active auditory landmarks: a melody played on a piano, a text read by a male voice, and a cicada. The choice of these sound samples was guided by the following criteria: they should be easy to discriminate on the basis of acoustic features (spectro-temporal content) as well as high level semantic content. Moreover they should be constantly active i.e., without periods of silence or abrupt changes. They were positioned on a horizontal plane at 1.60 m from the ground, i.e., on average slightly above the participant's sight level. The three landmarks were equalized so that their rms levels were identical (±1 dB) when the listener was located at the center of the exploration area. An auditory border delineated the exploration area. Whenever the participant crossed the limits of the exploration area a non spatialized sound of wind was rendered and the auditory landmarks were muted. In this case, the participant was instructed to come back inside the area whereupon she/he would be again immersed in the virtual environment. The hidden auditory target was located in a 60 × 60 cm zone. When walking on this zone, the participant activated the non spatialized sound of a whistle.

### Experimental procedure

The experiment lasted 2 h and was composed of a practice trial, 6 acquisition trials, and 4 test trials, each respectively immediately followed by an additional acquisition trial. The experiment ended with a probe trial.

The participants were led blindfolded into the experimental room and remained blindfolded until the end of the experiment. In order to avoid the construction of any mental preset about the space in which they were to perform the task, participants were blindfolded before entering the experimental room. To acclimate the participants with using a locomotor mode without vision, the participants were guided around the room in a preliminary acclimatization phase, after which the experiment started.

#### Practice (1 trial)

The participant walked in the exploration area to get used to the system and the soundscape. She/he had to search for the hidden auditory target (whistle tone) which was only triggered when entering and standing in a small zone (60 × 60 cm) located within the exploration area. If the participant did not find the target within 2 min, the practice trial terminated and she/he was guided outside of the exploration area, whilst hearing a non-spatialized masking sound (rolling pebbles).

The participants were instructed that their task during the next trials of the experiment was to find a similar target that would be hidden in a different location. Furthermore, they were instructed that the target location would henceforward remain the same for each subsequent trial.

#### Acquisition phase (6 trials)

The task of the participant was to search for, find, and stand on an initially inaudible target on the arena floor. When the participant found and stood on the target, it became audible, but reverted to being inaudible should the participant moved off it. As soon as the participant had found the target, the trial ended: the target sound and the auditory landmarks were then switched off and replaced with the non-spatialized masking sound (rolling pebbles) that was played until the commencement of the next trial.

Between each trial, the participant was randomly walked around in the experimental room to prevent any knowledge of the surrounding space. For each trial, lasting a maximum of 3 min, the participant started the exploration from a different entry point.

#### Test phase (4 tests)

For the test phase, the participant was informed that the location of the hidden target was identical to that of the hidden target in the acquisition phase, but that some aspects of the auditory landscape will have been changed from trial to trial.

There were four different conditions for this phase. In the first condition, the most proximal auditory landmark (cicada) was removed, therefore modifying the geometrical configuration of the landmarks (a line rather than a triangle), thus allowing for an evaluation of the participant's reliance on both the triangular configuration and the most proximal landmark (Test 1—Removal). In the second condition, all three auditory landmarks were rotated with respect to the exploration area and the location of the hidden target. As such, the distance relations between the exploration area, the hidden target, and the landmarks were all modified, whereas the geometrical configuration of the landmarks remained the same (Test 2—Rotation). In the third condition, the positions of two of the auditory landmarks (cicada and piano) were switched, while the third one remained unchanged (Test 3—Switch). In the fourth one, the auditory landmarks were unchanged, but the shape of the exploration area perimeter was modified (Test 4—Perimeter). Each of the test trials (capped at 3 min) was immediately followed by the initial configuration used in the acquisition trials in order to reaffirm the participant's familiarity with the original soundscape.

#### Probe

The final trial was a probe trial that had no more hidden target: the participants were regardless given the same, now impossible, task of finding the hidden target. This trial ended automatically after 2 min.

#### Debriefing

At the end of the experiment, the participants were guided blindfolded outside of the experimental room. They were then asked to draw a map of the soundscape that they had learnt and to comment on the experience.

### Data analysis

As the participants explored the environment, the position and orientation of their heads were recorded on average every 60 ms (15 Hz) and subsequently used to calculate their paths taken during the different phases of the experiment (Figure 1).

From these recordings, we assessed search time, i.e., the time to reach the hidden target, path length, and boundary crossings for all the trials. In order to study the effect of the tests, the following performance measures were also calculated:

1. Percent quadrant time: Amount of time participants searched in a virtual quadrant (i.e., 25% of total exploration area).
2. Boundary crossing per quadrant: Number of times the participant crossed the boundary of the exploration area per virtual quadrant.

For the tests and probe trial we computed the spatial distribution of the time elapsed in the exploration area, i.e., the percentage of time spent in a given location.

## Results

**Figure 2:**
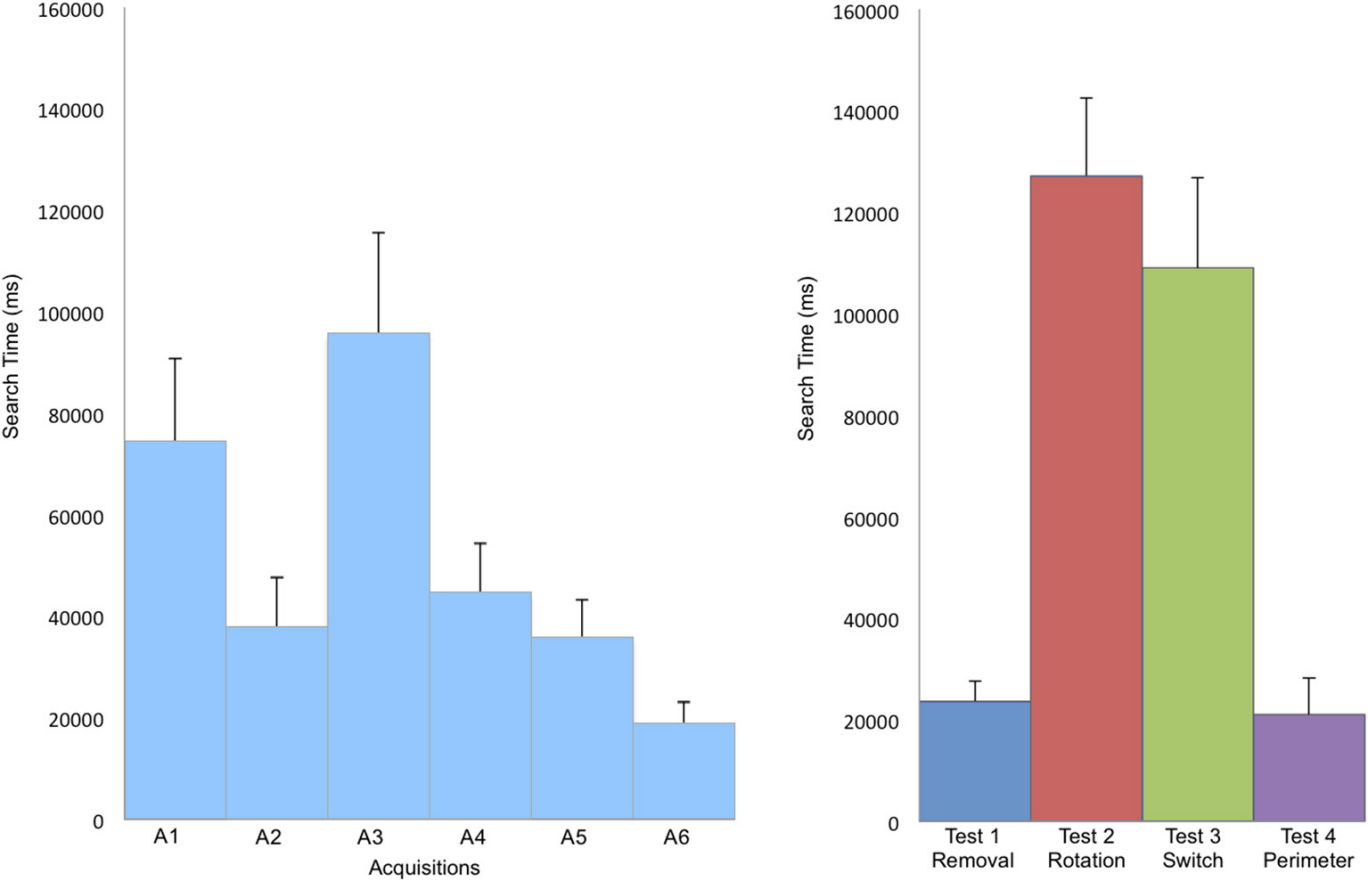
**Mean search time in ms to find the target during acquisition (A) and in the tests (B)**. Search time significantly decreases during the learning phase of the acquisition trials. Performances decreased when the landmarks were rotated (Test 2—Rotation) and when two landmarks were inverted (Test 3—Switch). Error bars represent the standard error of means.

There was a significant reduction of search time in finding the target across the 6 acquisition trials [repeated measures ANOVA, *F*_(5, 110)_ = 2.86, *p* = 0.02], indicating that participants had learnt how to find the hidden target (Figure 2). The number of boundary crossings and total length of the path covered followed the same pattern, diminishing with an increase in learning (Table 1).

**Table 1:** **Parameters of level of performance during the different phases of the experiment**.

		**Path length in m ± *SD***		**Boundary crossings ± *SD***	
**ACQUISITION PHASE (3 mn max)**					
	Trial 1	32.6	24.4	7.5	8.5
	Trial 2	17	17.7	3.2	5.2
	Trial 3	39.6	29.3	7.5	6.5
	Trial 4	19.8	12.8	3.6	2.7
	Trial 5	15.4	10	1.8	1.3
	Trial 6	8.9	9.1	0.9	1.9
**TEST PHASE (3 mn max)**					
	Test 1—Removal	10.2	7.8	1.5	2.1
	Test 2—Rotation	55.7	22.2	11.7	4.7
	Test 3—Switch	44.7	23.1	7.7	5.8
	Test 4—Perimeter	8.3	7.6	1	1.3
Probe (2 mn)		58.7	21.9	8.6	3.8

*Boundary crossings represent the number of times participants walked across the limits of the exploration area, triggering the sound of wind*.

**Figure 3:**
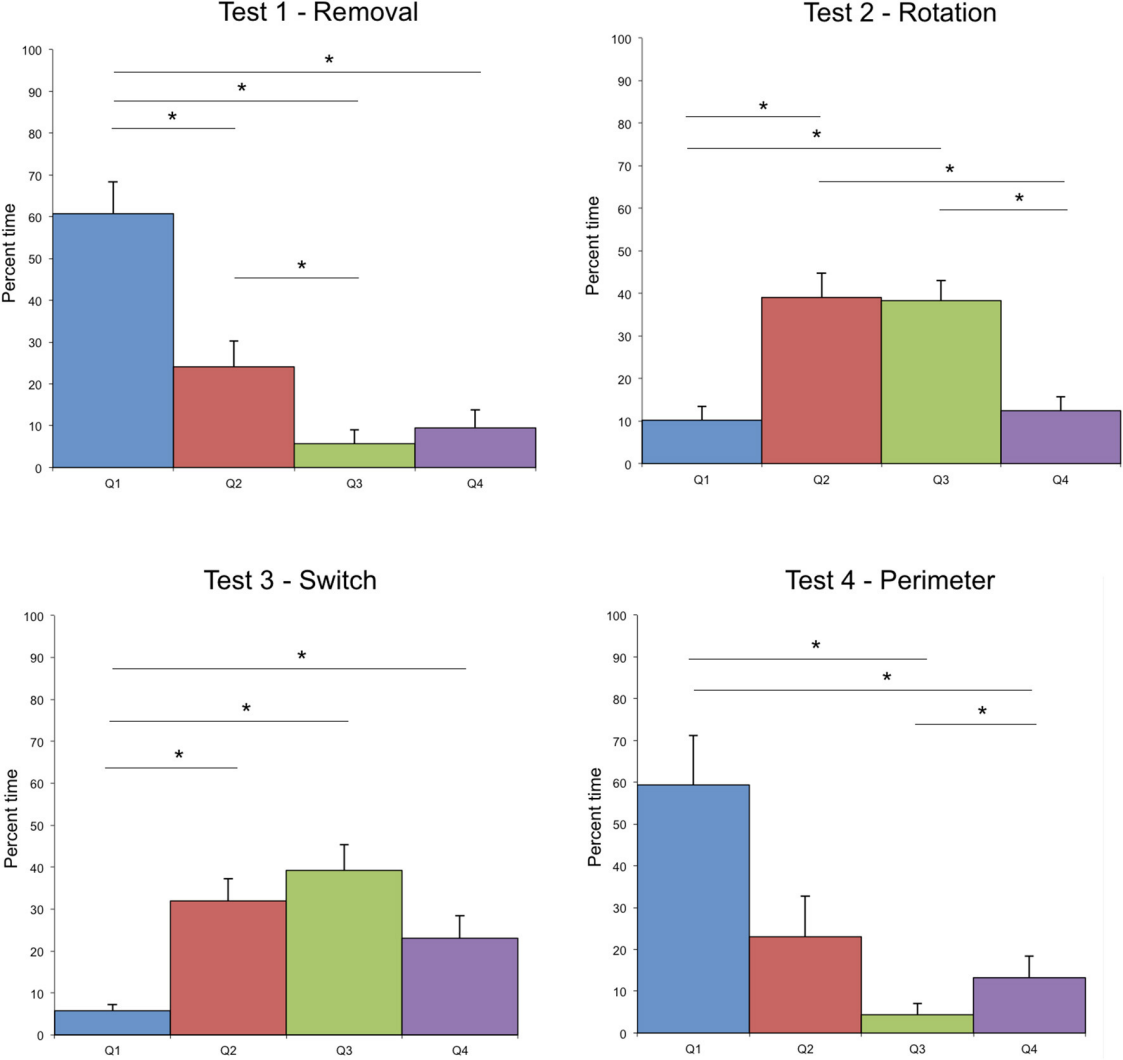
**Distribution of mean time spent in each quadrant of the exploration area in the four different tests**. Error bars represent the standard error of means, ^*^indicates significant differences. Test 2—Rotation, Wilcoxon signed rank test indicates differences between Q1 and Q2, *z* = 2.4, *p* = 0.02; Q1 and Q3, *z* = 2.7, *p* < 0.01; Q2 and Q4, *z* = 2.5, *p* = 0.01; Q3 and Q4, *z* = 2.6, *p* < 0.01. Test 3—Switch, Wilcoxon signed rank test indicates differences between Q1 and Q2, *z* = 2.9, *p* < 0.01; Q1 and Q3, *z* = 2.8, *p* < 0.01; Q1 and Q4, *z* = 2.7, *p* < 0.01.

The search time was different according to the modifications tested during the test (see Figure 2). The analysis of the virtual quadrants in the tests contrasted bias for the target location with other equivalent locations in the total area (Figure 3). Wilcoxon signed rank test indicated that in test 1—Removal (removal of a landmark), participants spent more time in the first quadrant (Q1), in which the target was located, than in the other quadrants (Q2, *z* = 2.49, *p* = 0.01; Q3, *z* = 2.8, *p* < 0.01; Q4, *z* = 2.67, *p* < 0.01). The absence of the closest landmark to the hidden target in Q1 did not seem to have a strong impact on their performance, as indicated by the mean search time. This suggests that the closest landmark to the target was not used as a beacon, but that the two other landmarks were equally used to localize the position of the target.

In test 2—Rotation (rotation of the 3 landmarks in relationship to the exploration area and to the hidden target) and test 3—Switch (inversion of two landmarks), the search time was much higher than in the two other tests. In both tests, both Q2 and Q3 were visited above the chance level: participants spent most of their time in those quadrants looking for the hidden target.

**Figure 4:**
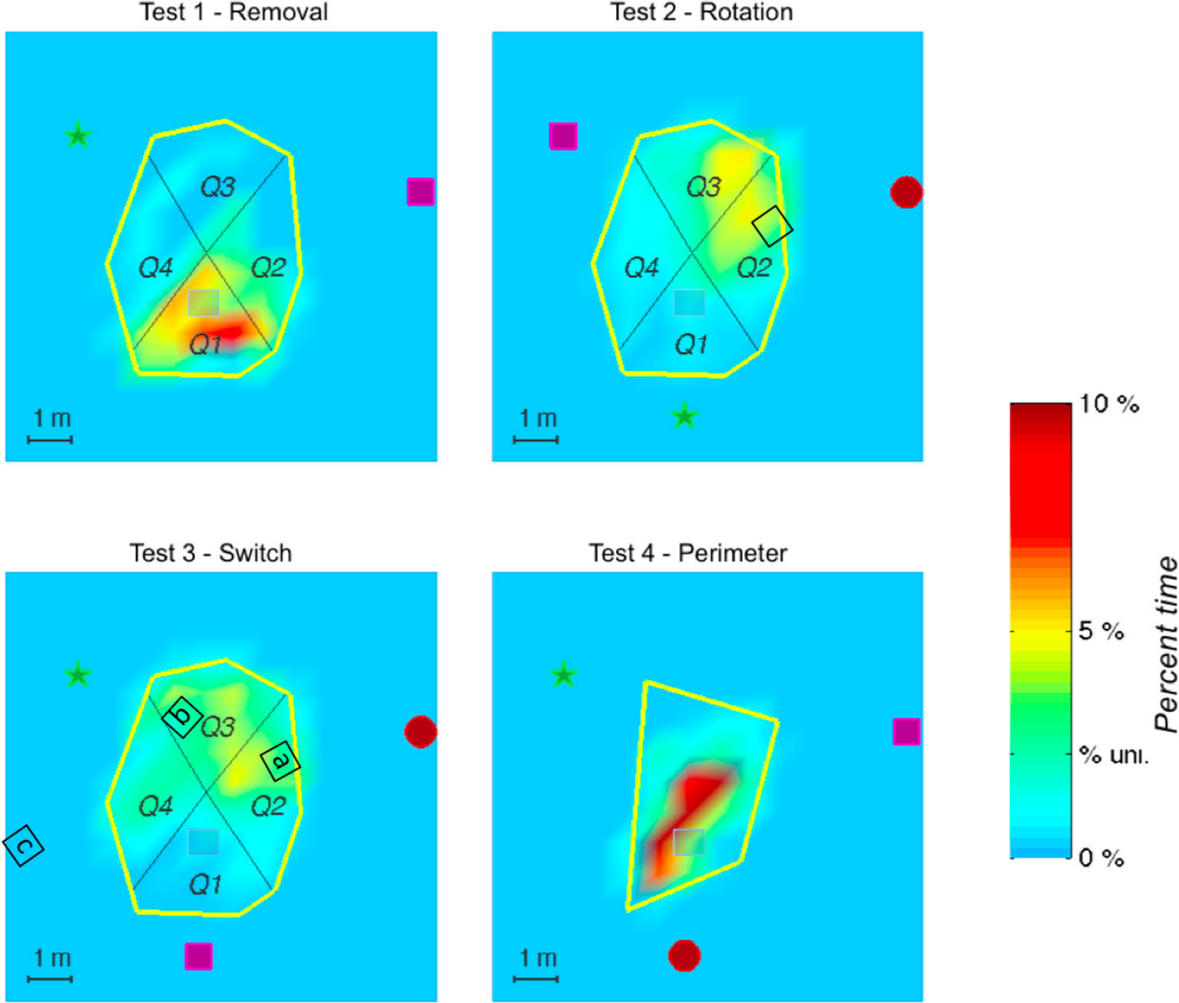
**The figure shows the spatial distribution of the time elapsed in the exploration area until the target was found and averaged on all subjects**. Color shadings represent percentage of time spent in a given location. The tick label “% uni.” on the scale corresponds to the hypothesis of a uniform spatial distribution. The actual position of the target is represented in gray. The red spot represents the cicada landmark, the green star represents the voice landmark, and the purple square represents the piano landmark. In Test 2—Rotation (landmarks rotation) and Test 3—Switch (inversion of 2 landmarks), the black squares represent different hypothetical positions of the target, corresponding to what could be tested by the participants according to their search strategy relative to the initial landmark configuration (angle and distance). Note that in Test 3—Switch, several possibilities could be explored by the participants, according to the characteristics of the auditory environment that was guiding the search (a- preservation of angle between landmarks with distance with cicada as a main cue, b- preservation of angle between landmarks with distance with piano as a main cue, c- preservation of angle between the piano and the voice, ignoring that the cicada is in the back instead of in front). If distance and angle to the voice landmark only were respected, while inversions of cicada and piano ignored, the target would remain located at the same place. The geometrical configuration of the landmarks seems to have been a strong cue in the search, whatever the test situation, as indicated by the heat maps.

In test 2—Rotation, the manner in which the paths are distributed, clustered in Q2 and Q3, indicates that it is not a singular landmark that serves as a navigational beacon. It is rather the relationship between landmarks, put into evidence by the preserved geometry of the landmarks, and the respective distance between the cicada landmark and the target that seems to serve as the primary strategy (see Figure 4).

In test 3—Switch, only Q1 was significantly different from the 3 other quadrants, which were equally visited, indicating an extension of the area of searching in this test. Indeed, according to the search strategy adopted, several solutions could be investigated by the participant to find the target (Figure 4). One of them would lead directly to the target (preservation of distance with the voice landmark together with a preservation of the geometrical organization of the 3 landmarks). This might account for the extension of the search area, and for the shorter search time than in test 2—Rotation.

In test 4—Perimeter (modification of the perimeter of the exploration area), the first quadrant was not significantly more visited than Q2 (*z* = 1.5, *p* = 0.1) and Q2 and Q3 were only marginally significantly different (*z* = 1.8, *p* = 0.08). This might be due to the efficiency of the participants in finding the target in this test, which permitted the participants to maintain the same search pattern across the quadrants. It seems that the modification of the perimeter of the exploration area did not impair the search strategy of the participants (see Figures 1, 2).

The analysis of boundary crossing per quadrant led to the same observations than with percent quadrant time, except in test 2—Rotation during which the amount of boundary crossings were slightly higher in Q2 than in Q3 (*z* = 1.9, *p* = 0.06). In this test, the cicada landmark was located in front of Q2, and was more distant to the exploration area than during the acquisition phase. It is possible that participants crossed the boundary several times in this quadrant when attempting to walk toward the cicada, usually the most proximal to the target in the acquisition phase configuration.

**Figure 5:**
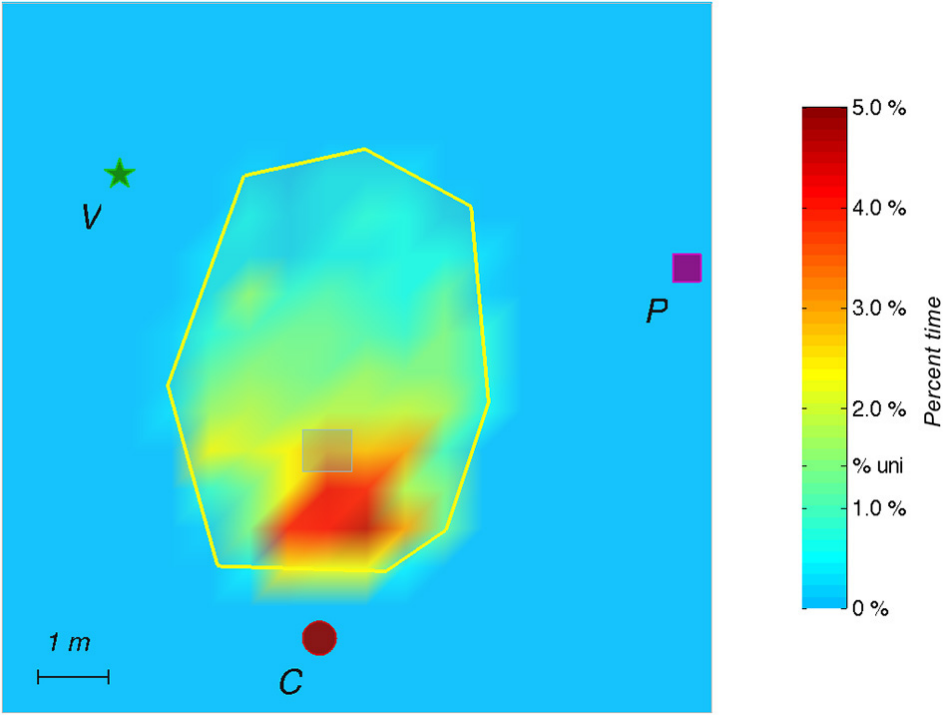
**During the Probe, the auditory target is removed without the subject being aware of it**. The figure shows the spatial distribution of the time elapsed in the exploration area (2 min) and on all subjects. Color shadings represent percentage of time spent in a given location. The tick label “% uni.” on the scale corresponds to the hypothesis of a uniform spatial distribution. The red spot represents the cicada landmark, the green star represents the voice landmark, and the purple square represents the piano landmark.

For the probe trial (without the hidden target), the heat maps indicate that participants most extensively searched near the target's supposed location (Figure 5).

### Debriefing

After the experiment, participants were asked to draw a map of the environment, marking the auditory landmarks and commenting on their strategy. All subjects accurately represented the triangular structure of the landmarks and its relationship to the target, with more or less precision but sometimes with stunning accuracy. Six participants drew instinctively a shape to define the exploration area: circular (4 participants) or rectangular (2 participants). The remaining five participants drew only the auditory sound sources and the target. No participants reported having built a visual mental imagery of the scene. Some participants were able to indicate what the modifications of the auditory landmarks were during the test phase. Only one participant suggested that the shape of the exploration area was different in the last test. One participant reported a path strategy to find the target, following a route along which the target would be found. Six participants described the usage of the angles between the landmarks, half of them also using the distance between the landmarks and the distance with the boundaries of the search area. One participant represented one of the landmarks position without being able to identify it, having forgotten its semantic content.

## Discussion

In this experiment, we wanted to explore whether spatial representation in blindfolded, normally sighted participants could be based on sensorimotor and auditory cues only. As indicated by the diminution of search time in the acquisition trials, and by the spatial distribution of the search paths in the probe test, participants had indeed learnt the spatial location of a hidden target without any visual information. The location of the target was surrounded by a set of landmarks. In test 1—Removal, we removed a landmark. In test 2—Rotation, because the geometrical configuration of the landmarks remained the same but was rotated with respect to the exploration zone, information about the target's enclosure by the landmark array conflicted with information about metric distance with the target. In test 3—Switch, we altered the adjacency relationship between landmarks from those that were learned. In test 4—Perimeter, only the geometry of the exploration area's boundaries was modified, leaving the angular relation between the location of the target and the set of landmarks untouched. The abilities that participants demonstrated strengthens the concept that spatial hearing has access to mechanisms for amodal spatial representations (Lakatos, 1993).

The test trials indicated that the representation of space learnt through audition and locomotion does not depend on auditory beacons. The cicada landmark was particularly salient because of its proximity to the boundary: we were thus expecting it to be used as a beacon, marking the nearby hidden target, and that other landmarks would provide information about one's current heading orientation. Should this have been the case, participants would have been impaired in the first test, in which the cicada landmark was removed. This rules out the usage of an egocentric strategy in which the spatial representation would be based on the relation between the location of the subject and the location of a single landmark.

### Auditory spatial representations depend on geometric relationships between auditory landmarks

The three auditory landmarks surrounding the hidden target in a triangular configuration were perceived as one triangle and not as three individual objects, as would be the case with visual objects. The geometry of the exploration array was not coded precisely since there was no coding of a “room,” but of individual relationships between walls (acoustically transparent boundary) and landmarks. In audition, there is no enduring representation of environment geometry that serves as a basis for reorientation. In visual environments, the geometric structure surface layout is said to persist much longer than the geometric relationships between distinct objects (Wang and Spelke, 2000). This might be an essential distinction in the contribution of these two sensory modalities to spatial knowledge. Whereas with vision, humans reorient themselves in accordance with the shape of the environment, they cannot do so with audition.

If the general shape of the room did not play a role in the representation of the auditory space, the boundary was a crucial cue (as suggested by the amount of boundary crossings in the different tests), just like in experiments with vision (Hartley et al., 2004). Subjects had to use distal landmark information as well as distance to the exploration area boundary to locate the hidden target. In the water maze tested with rodents, the maze walls are powerful cues used to locate the hidden platform even when they are transparent (Maurer and Derivaz, 2000). Boundaries of the environment play an important role in determining the place cells representation, and do so to an extent depending on their proximity (O'Keefe and Burgess, 1996).

### Direct access to an allocentric representation through audition

A major distinction has always been made between spatial representations linked to the observer (egocentric representations) and those that are independent from the observer (allocentric representations). Does this dichotomy exist for auditory perception? Because we can perceive the world only from our own position, it has been proposed that we create allocentric representations only through transformations of egocentric representations (e.g., Nadel and Hardt, 2004). However, this might not be true for auditory information, which might constitute a powerful input to the building of allocentric representations in real-world conditions.

Here we show that under the present experimental conditions, representations that underline place recognition were not purely egocentric: respective distances and directions of all features in the environment seemed to be the features that were looked for by the participants. Patterns of travel did not provide evidence that participants learnt to turn in specific directions at particular places. Only one participant reported at debriefing that his turning decisions depended on local representations of landmarks rather than on a global representation of the scene. However, this participant was perfectly able to draw an allocentric representation of the surface layout. Our results therefore favor the proposition of Holmes and Sholl (2005), stating that spatial information are stored in an allocentric reference system on which is superimposed an egocentric reference system depending on the position that is physically taken by the subject.

Humans may develop distinct types of environmental knowledge on the basis of different sensory cues (Yamamoto and Shelton, 2005). Because visual information is intimately linked to an eye-centered frame of reference, which is forward facing, it may provide an essential basis for landmark coding. Because auditory information allows for the perception of objects outside of reach and vision, they may provide not only egocentric (craniocentric) space representations, but also a direct access to allocentric coding of landmarks in space. Audition gives access to landmarks that are stable: they provide sensory inputs even when the subject turns his/her head from them. It is a crucial difference, which might be the key to the allocentric coding of an auditory scene. We therefore propose that auditory dynamic information allows for a direct coding of space in an allocentric manner.

### Movement to calibrate space

In the current experiment we could not test any specific hypothesis regarding sensorimotor information and auditory information and how they relate to each other. An additional condition would be to ask the participant to move passively in the auditory scene, e.g., by controlling his/her navigation through a joystick. We have started to test this condition, and preliminary results suggest that learning of the spatial location of the hidden target is impaired when there is an absence of any visual and sensorimotor information linked to self-movement. Further experiments are needed to understand how acoustic inputs are related to motor states and which parameters of the auditory-motor loop are relevant for the building of spatial representations.

Because spatial audition is mainly studied in humans with a fixed position in space, the possibilities of human spatial audition to encode space are often underestimated. There is a gap in the literature on spatial cognition in humans and in animals. In rodents, major categories of spatial cells have been discovered (place cells, head direction cells, grid cells and boundary cells), each of which having a characteristic firing pattern that encodes spatial parameters relating to the animal's current position and orientation (see Hartley et al., 2013 for a recent review). For these experiments, the animals move freely in an arena, integrating sensorimotor cues together with other sensory cues. In humans, it is very seldom that sensorimotor integration is taken into account when studying spatial cognition. This is also the case when studying spatial auditory cognition, which is mainly studied in deprivation of other senses. In spite of the rarity of studies in audition taking movement into account, it has already been suggested that auditory localization processes combine the acoustic input with head position information to encode targets in a body-centered frame rather than an external visual frame of reference (Goossens and van Opstal, 1999), and that dynamically varying acoustic cues are adequately processed to build a representation in world coordinate (Vliegen et al., 2004).

The role of visual information to calibrate auditory spatial cognition has been underlined by many (e.g., Hofman et al., 1998; Zwiers et al., 2003; Sarlat et al., 2006; King, 2009). More recently, the role of sensorimotor calibration of audition emerges as very significant (Aytekin et al., 2008; Boyer et al., 2013; Carlile and Blackman, 2013; Carlile et al., 2014). Here we provide data attesting that when humans can use sensorimotor information, their spatial map of an auditory space is very accurate. When perceived in movement, auditory information is probably of paramount importance to sense space, even in normal sighted humans. The motor calibration of auditory space connects the ear to the body and to the space around us.

### Conflict of interest statement

The authors declare that the research was conducted in the absence of any commercial or financial relationships that could be construed as a potential conflict of interest.
